# Individual finger movement decoding using a novel ultra-high-density electroencephalography-based brain-computer interface system

**DOI:** 10.3389/fnins.2022.1009878

**Published:** 2022-10-19

**Authors:** Hyemin S. Lee, Leonhard Schreiner, Seong-Hyeon Jo, Sebastian Sieghartsleitner, Michael Jordan, Harald Pretl, Christoph Guger, Hyung-Soon Park

**Affiliations:** ^1^Department of Mechanical Engineering, Korea Advanced Institute of Science and Technology (KAIST), Daejeon, South Korea; ^2^g.tec Medical Engineering GmbH, Schiedlberg, Upper Austria, Austria; ^3^Institute for Integrated Circuits, Johannes Kepler University, Linz, Austria

**Keywords:** BCI, EEG, high-density EEG, ultra-high-density EEG, machine learning, finger decoding, motor execution

## Abstract

Brain-Computer Interface (BCI) technology enables users to operate external devices without physical movement. Electroencephalography (EEG) based BCI systems are being actively studied due to their high temporal resolution, convenient usage, and portability. However, fewer studies have been conducted to investigate the impact of high spatial resolution of EEG on decoding precise body motions, such as finger movements, which are essential in activities of daily living. Low spatial sensor resolution, as found in common EEG systems, can be improved by omitting the conventional standard of EEG electrode distribution (the international 10–20 system) and ordinary mounting structures (e.g., flexible caps). In this study, we used newly proposed flexible electrode grids attached directly to the scalp, which provided ultra-high-density EEG (uHD EEG). We explored the performance of the novel system by decoding individual finger movements using a total of 256 channels distributed over the contralateral sensorimotor cortex. Dense distribution and small-sized electrodes result in an inter-electrode distance of 8.6 mm (uHD EEG), while that of conventional EEG is 60 to 65 mm on average. Five healthy subjects participated in the experiment, performed single finger extensions according to a visual cue, and received avatar feedback. This study exploits mu (8–12 Hz) and beta (13–25 Hz) band power features for classification and topography plots. 3D ERD/S activation plots for each frequency band were generated using the MNI-152 template head. A linear support vector machine (SVM) was used for pairwise finger classification. The topography plots showed regular and focal post-cue activation, especially in subjects with optimal signal quality. The average classification accuracy over subjects was 64.8 (6.3)%, with the middle versus ring finger resulting in the highest average accuracy of 70.6 (9.4)%. Further studies are required using the uHD EEG system with real-time feedback and motor imagery tasks to enhance classification performance and establish the basis for BCI finger movement control of external devices.

## 1 Introduction

Brain-Computer Interface (BCI) technology employs brain activity and the underlying neural information to control external equipment such as computers, wheelchairs, and prostheses without needing to physically move body parts (Belkacem et al., 2020). This technology can help people suffering from severe motor disabilities to communicate with the outside world and indirectly restore motor function (Wolpaw et al., 2002). The applications for complete or partially paralyzed patients, such as stroke patients (e.g., Hemiplegia), have been widely investigated for decades (Harkema et al., 2011; Bundy et al., 2017; Guger et al., 2017; Bai et al., 2020; Sebastián-Romagosa et al., 2020; Bhatia et al., 2021).

Rehabilitating motor functions of the hand, especially fingers, is essential for improving activities of daily living (ADLs) for people who experience upper limb motor impairment. Finger movements are required to manipulate tools, grab and move objects, and make signs, which are functions repeatedly used and indispensable (Landsmeer, 1962; Jones and Lederman, 2006; Mawase et al., 2020). Therefore, to better understand and rehabilitate the finger-related motor cortex, a considerable amount of research has been conducted (Kubánek et al., 2009; Acharya et al., 2010; Yanagisawa et al., 2011; Flamary and Rakotomamonjy, 2012; Liang and Bougrain, 2012; Hotson et al., 2016). For example, Acharya et al. (2010) and Kubánek et al. (2009) observed a focused brain activation region during hand grasp and thumb flexion, respectively. Hotson et al. (2016) and Gruenwald et al. (2019) classified individual finger tapping with accuracies above 95% using high-density electrocorticography (ECoG) grids.

These results are outstanding but cannot be generalized because most studies used invasive methods such as ECoG to acquire brain activity. Since ECoG acquires electroencephalographic signals directly from the cerebral cortex, it provides high spatio-temporal resolution and signal-to-noise ratio (SNR), producing excellent results in detecting single finger movements. However, invasive methods require clinical surgery to place the subdural electrode grids directly on the surface of the cortex.

This limitation demands using non-invasive methods, such as electroencephalography (EEG). EEG acquires electrophysiological signals from the scalp. Since EEG does not require clinical surgery and offers a high temporal resolution with low price and high portability, it is the most widely used among various brain signal acquisition methods (Mason et al., 2007). Moreover, these characteristics empower EEG-based BCI systems to be used as real-time closed-loop applications, the final goal of BCI application systems.

However, EEG systems have several limitations. It has low spatial resolution caused by common EEG electrodes’ large layout and size and its susceptibility to artifacts that often originate from movement and electromyographic (EMG) activity (Al-Fahoum and Al-Fraihat, 2014). Due to its limitations, the current state-of-the-art EEG systems only allow the distinction of gross movements. For example, research was done to discriminate hand grasp and release (Lange et al., 2016), right and left-hand movements (Pfurtscheller et al., 1997), upper-limb movements (Ofner et al., 2017), and lower-limb movements (Mejia Tobar et al., 2018).

Decoding more precise movements, such as individual finger movements, is highly challenging for conventional EEG systems. Quandt et al. (2012), Stankevich et al. (2016), and Bera et al. (2019) decoded individual finger movements using frequency bands under 40 Hz. They resulted in an average classification accuracy of 60, 50, and 43%, where the chance level was 33, 25, and 25%, respectively. Liao et al. (2014) classified finger pairs and reached 59 and 77% accuracy using the mu frequency band (8–12 Hz) and broadband features, respectively.

**FIGURE 1:**
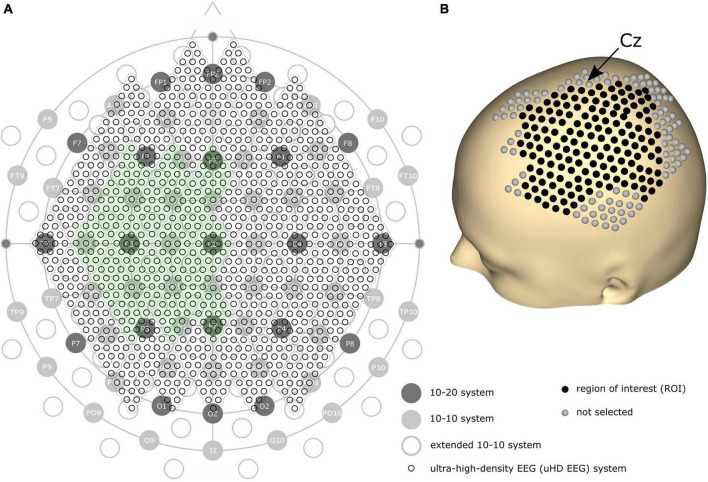
**(A)** Comparison of the 10–20, 10–10, extended 10–10, and ultra-high-density electroencephalography (uHD EEG) systems–black circles with labels illustrate the 10–20 system; gray filled circles with electrode names show the positions of the original 10–10 system; gray non-filled circles indicate the extended positions of the 10–10 system; the entirety of the small black circles show the 1,024 uHD EEG system. The green-colored area indicates the electrode positions used in this study (256 electrodes out of 1,024 electrodes). **(B)** 3D model of the human scalp with the marked electrode positions. Cz is pointed out with an arrow. Each sphere corresponds to a single electrode, where black-colored electrodes indicate the region of interest for the classification.

We assume a higher spatial resolution can improve the performance of EEG on precise tasks. Since denser electrodes can increase the spatial resolution, the amount and density of EEG electrodes have historically been steadily increasing. The first standard placement of EEG electrodes was proposed by Jasper (1958) with the definition of the 10–20 electrode system using 21 electrode locations over the whole scalp (see Figure 1A, dark gray circles). In 1985, the extended version of the 10–20 system, the 10–10 system, was proposed using 74 electrode locations (see Figure 1A, light gray circles), which is currently accepted as the standard by the American Electroencephalographic Society (Chatrian et al., 1985; Sharbrough, 1991). Based on the 10–10 system, another extended version, the extended 10–10 system, was proposed using 128 electrodes [(Oostenveld and Praamstra, 2001); see Figure 1A, gray non-filled circles]. Several high-density EEG systems are available commercially, primarily using from 160 to 256 channels. However, some devices contain electrodes placed on the cheeks and neck that record less motor-related brain activity. Therefore, more electrodes do not necessarily result in high motor classification performance.

Can’t the number of electrodes further increase? Yes, but only by breaking the convention that the electrode position should follow the international 10–20 system. Several studies have demonstrated that narrowing down the inter-electrode distance under 2 cm–commonly and widely used inter-electrode distance–is beneficial since it provides additional neural information (Freeman et al., 2003; Odabaee et al., 2013; Petrov et al., 2014; Robinson et al., 2017). For this reason, some recent studies explored denser electrode layouts in visual and auditory contexts (Robinson et al., 2017; Chamanzar et al., 2021; Narayanan et al., 2021). Here, we define a novel array formatted dense electrode layout (see small black circles in Figure 1A) as ultra-high-density EEG (uHD EEG). Theoretically, 1,024 electrodes can be placed over the whole scalp following the electrode size and position of this layout.

In order to achieve high SNR, active and wet electrodes are preferred over passive and dry electrodes. Active and wet electrodes use pre-amplifiers to amplify the acquired signal and conductive gel to bridge the scalp and electrodes (Mathewson et al., 2017). However, if the uHD EEG system consists of wet electrodes and is placed with a cap or a similar stretchable structure, as is the case for conventional EEG, the conductive gel of adjacent electrodes may touch due to its proximity (Rashid et al., 2020). This problem can be solved by changing the attachment method from a cap type to a medical adhesive. The conductive gel can be separated into holes using medical adhesives that have resections at each electrode site, consequently providing consistent low impedance and electrode separation.

In this study, we used a newly proposed uHD EEG, g.Pangolin (g.tec Medical Engineering GmbH, Schiedlberg, UA, Austria), and explored the performance by decoding individual finger movements of one hand. To the best of our knowledge, no experimental study using such uHD EEG in motor tasks has been reported (while visual and auditory tasks have been explored). The electrode grids were directly attached to the scalp using medical adhesives. Five subjects participated in the experiment and extended individual fingers according to a visual cue. Collected data were preprocessed, and features were extracted for the mu (8–12 Hz) and beta (13–25 Hz) frequency bands. In order to assess the performance of the system, topography plots were generated for the band power features, and a linear support vector machine (SVM) classifier was used for pairwise finger classification.

## 2 Materials and methods

### 2.1 Subjects

Five healthy subjects [S1–S5, one female and four males, age: 28 (7) years in mean (SD)] participated in the experiment. Subjects S2–S5 were right-handed, whereas subject S1 was left-handed. The participants did not receive any prior training regarding this experiment. The study was approved by the Institutional Review Board of Korea Advanced Institute of Science and Technology (KH2018-127). All participants gave written consent forms about recruitment prior to data collection.

### 2.2 Experimental protocol

**FIGURE 2:**
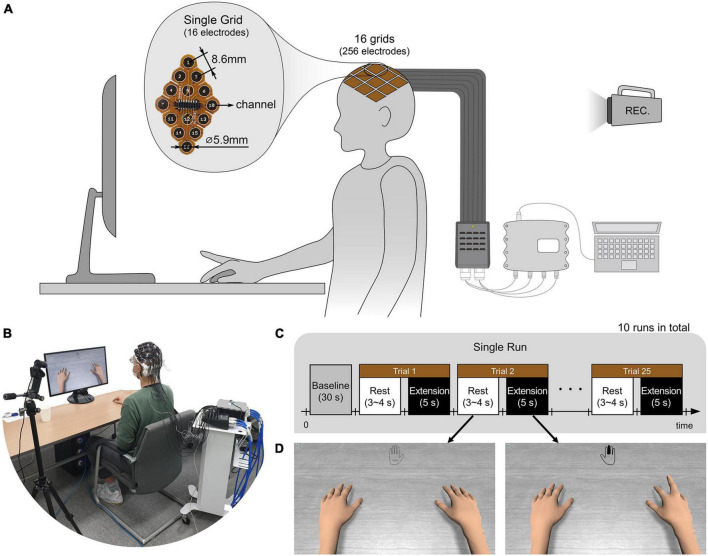
**(A,B)** Illustration and picture of overall system setup. The ultra-high-density electroencephalography (EEG) system is placed over the contralateral sensorimotor cortex of the subject’s scalp and connected *via* pre-amplifiers and HDMI cables to an interface box. Further, the 256-channel amplifier g.HIamp (g.tec Medical Engineering GmbH, Schiedlberg, UA, Austria) is connected through USB to the acquisition computer. A camera recorded the whole experiment. **(C)** The subject extended one of the five fingers and rested according to the experimental paradigm. A single run consisted of 25 trials with five trials per finger. 10 runs were conducted for each subject. **(D)** According to the paradigm, the monitor showed instruction cues and the hands of an avatar. The instruction was provided at the top center of the monitor with the colored finger the subject should extend. The right picture shows an example of when the middle finger should be extended. During the rest period, no finger was colored, and the hand was grayed out as shown in the left picture. As ideal visual feedback, the hand avatar simultaneously performed the finger extension as the subject should do.

Prior to the experiments, the subjects’ heads were shaved and cleaned with medical alcohol to attach uHD EEG grids directly to the scalp. As in Figures 2A,B, subjects sat on a comfortable armchair with their dominant hand placed on the table and their palm facing down. Figure 2C shows the experimental protocol, with the whole experiment consisting of 10 sequential runs. Each run lasted roughly 5 min, and the subject could take a break between runs. A single run started with a 30 s baseline period, followed by alternating rest and extension periods. During the rest period, the left picture of Figure 2D was shown on the monitor, and the subject could relax, swallow, or blink their eyes. The rest period lasted randomly between 3 and 4 s to prevent the subjects from adapting to the protocol. During the 5 s extension (i.e., task) period, the subject extended the finger according to the instruction cue and maintained it without moving any other part of their body. The instruction cue reflected a hand for which a single finger was colored and displayed in the monitor’s top-center (see Figure 2D right picture, middle finger extension). All fingers were pseudo-randomly cued five times in each run, resulting in 25 finger extensions per run. The hand avatar provided ideal (i.e., non-online feedback) visual feedback by imitating the finger rest and extension movements (see Figure 2D). Thus, the visual feedback can be thought of as an instruction that subjects should follow.

### 2.3 Ultra-high-density electroencephalography system

Brain activity was measured using a novel uHD EEG system. The g.Pangolin system can be considered uHD due to its spatial resolution. A single electrode grid consists of 16 electrode points with a diameter of 5.9 mm and a center-to-center spacing of 8.6 mm. In this study, 16 electrode grids (256 electrodes) were attached over the contralateral sensorimotor cortex of the subject’s scalp (see Figure 1A, the green-colored area). Instead of using the 1,024 electrodes, only using electrodes over the contralateral sensorimotor cortex would improve the performance of motor control when channel selection algorithms or feature weights are used together (Arvaneh et al., 2011; López-Larraz et al., 2014). Figure 1B shows the electrode position on the subject’s scalp. The black-colored electrodes (158 electrodes) reflect the region of interest (ROI), while gray-colored electrodes were excluded from the classification step for this motor movement task. We removed the most anterior, posterior, central, and temporal electrodes to reduce the number of features during classification while retaining electrodes over the subjects’ sensorimotor hand area.

The electrode grids were attached sequentially in the downward direction laterally of the head, starting with the first electrode grid at the Cz position. Since the electrode grid is diamond-shaped and attached to share each other’s side, the sensor locations were pre-defined only with the starting point and the direction. The ground electrode from the uHD EEG system was placed on the left mastoid for all experiments.

Figures 2A,B show the system setup and data acquisition procedure for the conducted experiments. The electrode grid was attached to the skin using medical adhesives that insulated the electrode sites. The resection in the adhesive was filled with conductive paste, Elefix (Nihon Kohden Corporation, Japan), to ensure optimal skin contact and low impedance at the electrode-skin junction. For better signal quality and higher SNR, a pre-amplifier is used. Through this stage, the raw EEG recorded from the scalp is amplified with a gain of 10. An interface box connects the high-resolution electrode grids with the pre-amplifier and the biosignal amplifier. The pre-amplifier is connected to the interface box using a micro-HDMI to HDMI cable. The connection to the g.HIamp is established with a 65-pin interconnect cable with LEMO connectors to transmit 64 channels and Ground (GND). The interface box allows 256 channels to be recorded with the g.HIamp biosignal amplifier using 4 LEMO cables.

Data acquisition and paradigm presentation were performed with g.HIsys Professional (g.tec Medical Engineering GmbH, Schiedlberg, UA, Austria) running under MATLAB/Simulink (The MathWorks, Inc., Natick, MA, USA). Both the visual cue and avatar were presented using Unity (Unity Technologies, San Francisco, CA, USA). The signals were recorded with a sampling frequency of 600 Hz, and the cue information was synchronously recorded with the EEG data in real-time.

### 2.4 Data processing

**FIGURE 3:**
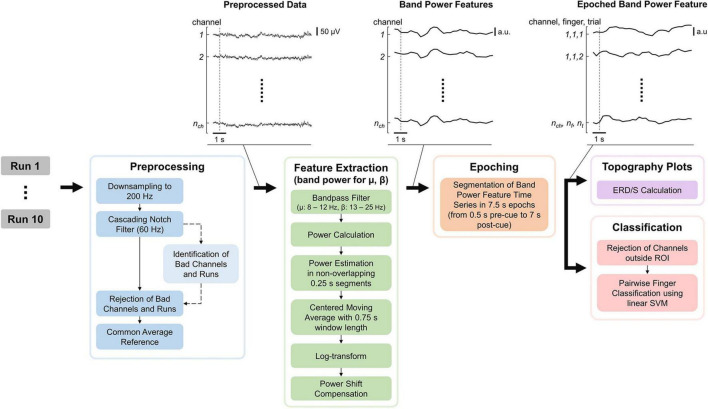
Electroencephalography (EEG) processing pipeline consisting of preprocessing, feature extraction, epoching, topography plots, and classification. “Identification of Bad Channels and Runs” returns only the information which channel(s) and run(s) are bad (dashed arrow) while leaving the EEG data (solid arrows) unchanged. The data were visualized after each processing step for an example dataset with the dashed line indicating the start of the first trial. n_ch_, n_f_, and n_t_ are the number of channels, fingers, and trials, respectively.

The EEG processing pipeline consisted of Preprocessing, Feature Extraction, Epoching, Topography plots, and Classification of the data and was implemented in MATLAB (see Figure 3). MATLAB commands are provided to improve reproducibility and are formatted in *italic*. The EEG processing pipeline is quasi causal (except for the bad channel identification and moving average), deterministic and computation cost was kept low as the pipeline is intended to be used in real-time and online BCI experiments. Note that both bad channel identification and moving average could however be implemented in a causal manner. The recorded EEG is unlikely to be affected by ocular (e.g., eye movements and eye blink) and movement artifacts as the electrodes did not cover the frontal lobe, and subjects only moved their fingers during the tasks.

#### 2.4.1 Preprocessing

The raw EEG recordings were first down-sampled from 600 to 200 Hz (*resample*) and notch-filtered at 60 Hz and its harmonics using a notch-filter cascade. 4th-order Butterworth filters were utilized, using *butter* to calculate filter coefficients and apply the filter coefficients to the signal. Next, bad channels and runs were identified based on EEG data during the task period. Note that this step only provides the identified channels and runs but leaves the previously notch-filtered EEG data unchanged (see Figure 3). First, data were common average referenced and filtered using a band-pass filter from 8 to 25 Hz. Second, for each channel, the mean power was computed by subtracting their respective mean, calculating the power by the squaring of each time sample, and calculating the mean power over time. Third, the mean channel powers were log-transformed to improve Gaussianity and z-transformed. Finally, channels with a z-score greater than 6 were considered bad, and runs in which more than 10% of channels are bad were removed. Furthermore, a disjunction was applied to bad channels over the remaining runs (i.e., if a channel is considered bad in one run, it is considered bad in all runs). Bad channels found using this procedure were removed from the notch-filtered EEG data, which were finally common average referenced.

#### 2.4.2 Feature extraction and epoching

The features extracted are band power features for the mu and beta bands, as these frequency bands are associated with motor functions (Miller et al., 2010; Cheyne, 2013). The EEG data were band-pass filtered for the respective frequency band, and the power was calculated by squaring each time sample. Then the power was estimated in non-overlapping 0.25 s segments by averaging the power samples and applying a centered moving average with a 0.75 s window length. A centered moving average was used instead of a causal moving average in order to make the interpretation of results easier, as the latter would introduce time delays. Again, band power features were log-transformed to improve Gaussianity because non-log-transformed band power features are Chi-squared distributed. Finally, a power shift compensation was applied, subtracting the mean band power over the last 25 s. This is important as the band power of any channel can have slow drifts over time which will bias the classifier (see Supplementary material–Supplementary Figure 2 and also investigated by Benwell et al. (2019). A window length of 25 s was chosen as it comprises multiple trials while still staying responsive. Specifically, choosing the window length too short would lead to removing task-related information. Finally, the band power features were epoched using 0.5 s pre-and 7 s post-cue.

High-gamma activity (70–170 Hz) was also investigated (see Supplementary material–Supplementary Figure 1 and Supplementary Tables 1, 2) as these features showed positive results in classifying individual finger movements (Liao et al., 2014). However, we did not find high-gamma activity to have any added value for visualization of activity patterns or classification.

#### 2.4.3 Topography plots

A 3D model of the MNI-152 template brain (Montreal Neurological Institute, Canada) was employed for visualization of the activation plots generalized over all subjects. 16 uHD EEG electrode grids were placed on the MNI-152 head using a custom montage creator software (g.tec Medical Engineering GmbH, Schiedlberg, UA, Austria). The resulting montage contained 256 electrodes located on the left hemisphere.

All subjects except subject S1 are right-handed. Therefore, the left hemisphere was selected for plotting the topographies over all subjects. In the case of subject S1, the sensor locations are redistributed by multiplying the x-axis coordinates by −1, which results in the same electrode layout mirrored on the sagittal plane having consistent plots.

The relative change in band power, commonly known as event-related desynchronization/synchronization [ERD/S; Pfurtscheller and Lopes da Silva (1999)], was calculated as:


$$\text{ERDS}\left[\text{n}\right]=\log\left(p\left[n\right]\right)-\frac{1}{\left|{𝒮}_{REF}\right|}\sum_{k\in {𝒮}_{REF}}\log\left(p\left[k\right]\right)$$

where *p* are the band power features and 𝒮_*REF*_ contains the time-discrete band power features within the reference period ranging from 0.5 to 0 s before the cue. While this may seem like a short reference period, note that a centered moving average was used in the feature extraction (see Figure 3). Therefore, each band power sample is related to the previous two-band power samples. Finally, mu and beta ERD/S during the average trial were illustrated in the topographies and reflect changes in dB.

Heatmaps based on ERD/S activation were generated according to Kubanek and Schalk (2015). For each electrode, the surface vertices closer than the Euclidean distance to the adjacent electrode were determined. An activation value was assigned to each vertex by convolving the activation of each electrode with a linear decay kernel. The kernel was chosen to reach zero at the Euclidean distance to the closest electrode. Finally, the activation values assigned to each vertex were summed up, and the vertices were colored accordingly. Bad channels identified during the preprocessing (see sections “2.4.1” and “2.4.2”) are excluded from the heatmap interpolation. Depending on the number of bad channels, the process of channel removal can result in irregular heatmaps with vacant sensor locations.

Spatial ERD/S dynamics were explored to investigate the spatial capabilities of the uHD EEG system. Focal activation plots from each finger extensions were created using the electrodes with the greatest ERD. Electrodes with the greatest ERD were selected by thresholding the mean ERD/S during seconds 1–2 post cue of the average trial. The threshold was set at the 2.5th percentile of all electrodes for the respective finger extension. In other words, electrodes which show greater ERD than 97.5% of all electrodes were selected. This selection results in ∼6 electrodes per finger extension.

Time-frequency maps were generated to investigate the temporal ERD/S dynamics. First, the power was estimated using the short-time Fourier transform (*spectrogram*) from 8 to 30 Hz at 1 Hz steps employing a 1 s Hamming window with 0.95 s overlap, resulting in a sampling frequency of 20 Hz. Second, the power samples were log-transformed and epoched using 1 s pre-and 7 s post-cue. ERD/S was computed with reference period 1 to 0 s before the cue. Finally, the ERD/S of the average trial was interpolated by a factor of 10 in time and frequency (*ndgrid, griddedInterpolant*) to improve image quality.

#### 2.4.4 Classification

Classification models were employed to investigate if the extracted band power features have predictive power. Specifically, band power features during the task period (i.e., 0 to 5 s) for electrodes contained in the ROI were classified at each time step. Each subject’s finger movements were pairwise classified using a linear SVM, resulting in 5(5–1)/2 = 10 two-class classification problems. The reasons for choosing a simple linear SVM (*fitcsvm*) are two-fold. It was chosen instead of more complex models as it is suitable for online BCI experiments due to its relatively short training times (Lotte et al., 2007, 2018; Rashid et al., 2020). Secondly, it is not vulnerable to numerical instabilities when the number of features exceeds the number of training data points (i.e., instances, trials, observations), compared to a linear discriminant analysis (LDA) which would need strong regularization. Similarly, common spatial patterns (CSP) and filter bank CSP (FBCSP) were not used as the optimal size and position of the CSP window would need to be optimized for using a cross-validation (CV) procedure. Furthermore, due to the high number of electrodes regularized CSP (rCSP) is likely necessary, adding additional optimization parameters (Lu et al., 2009; Lotte and Guan, 2011). Nonetheless, we are not implying that the approach chosen in this study is necessarily superior to CSP based approaches.

For the SVM, the kernel scale of a linear kernel was automatically selected by MATLAB using a heuristic approach, features were standardized and a box constraint of 1 was used. This box constraint is used for regularization and helps to prevent overfitting. Notably, the results reported here are based on SVM models without hyperparameter optimization to keep training times to a minimum. While not reported here, results obtained using hyperparameter optimization of kernel scale and box constraint were similar, which is in line with findings by Probst et al. (2019).

An *n*-times of *k*-fold CV framework was used to estimate the predictive power of the band power features, with *n* and *k* both being 10. In other words, all trials of all runs (i.e., recordings) were used in a 10-fold CV, which was performed 10 times leadings to different CV assignments in each of the 10 iterations and thus a more reliable estimation of the performance. The random seed (*rng*) was set to the respective iteration (i.e., 1 to 10) to make cross-folds, and thus results reproducible. 10-fold CV was used instead of a leave-one-out CV, because the latter provides unstable and biased model performance estimates (Varoquaux et al., 2017). Ten-times of 10-fold CV were used as 10 iterations already provide reliable estimates of model performance (Bouckaert and Frank, 2004). The model performance was defined as the mean classification accuracy over these 10 iterations, at the time point with highest mean classification accuracy. Note that one may also perform a leave-one-run-out CV in which one trains the model using all but one run and then uses the remaining run as the test data. The model performances obtained using this framework are slightly better than the ones obtained using 10-times of 10-fold CV and can be found in the Supplementary material (see Supplementary Table 3).

Empirical *P*-values were calculated to quantify the chance of the observed model performance being obtained by chance. These *P*-values were obtained using a permutation testing approach in which the dependence between the features and the target variable (i.e., class) is broken (i.e., null model). In these null models, the target variable is permuted (i.e., shuffled), and then the 10-times of 10-fold CV is carried out to estimate the null model performance. This was done 100 times, resulting in 100 model performances obtained under the null hypothesis of independence of features and the target variable. As these null model performances are normally distributed, a normal distribution was fitted to these data, and the *P*-value for obtaining a performance measure as extreme or more extreme than the observed one was computed. This approach results in similar *P*-values as the approach used by Ojala and Garriga (2009). They calculated the fraction of null models with equal or greater model performance than the observed one, except that *P*-values smaller than 0.0099 can be obtained.

Empirical *P*-values were calculated for the time point which showed the greatest classification accuracy in the observed model and for each of the 10 classification problems and the respective subject. Model performance was considered to be better than the chance for *P* < 0.05. The *P*-values computed and reported were corrected for multiplicity (i.e., multiple hypothesis testing) per subject using the Benjamini-Hochberg [i.e., false discovery rate (FDR)] procedure (Benjamini and Hochberg, 1995).

In order to investigate if the additional electrodes introduced by the uHD EEG system increase classification accuracy, electrode subsets reflecting the 10–10 and extended 10–10 system were selected. The electrode subsets were generated by selecting individual uHD electrodes which best fit the respective 10–10 and extended 10–10 system positions (see Figure 1A and Supplementary Figure 3). For these two electrode subsets the band power features were computed (see sections “2.4.1” and “2.4.2”) and their predictive power was estimated. Finally, the 50 model performances (5 subjects with their 10 pairwise classifications) for the uHD, 10–10 and extended 10–10 system were compared using a two-tailed Wilcoxon signed-rank test.

#### 2.4.5 Correlation between neighboring electrodes

A correlation analysis was performed to investigate to which extent neighboring electrodes provide identical information in the case of uHD EEG system, as well as the electrode subsets reflecting the 10–10 and extended 10–10 system. For all, but electrodes at the edge of the montage, neighboring electrodes were identified as the four-nearest neighbors according to their center-to-center Euclidean distance (see Figures 1A, 2A). Edge electrodes only served as neighbors as they are not enclosed by four-nearest neighbors. Note that only unique electrode pairs were allowed (e.g., electrode *e*_*i*_ is electrode $e_{j}^{\prime }s$ nearest neighbor and vice versa, resulting in one unique electrode pair). EEG data provided by the preprocessing step (see Figure 3) were band pass filtered between 1 and 30 Hz to contain the frequency components typically used in EEG experiments. Finally, the center-to-center Euclidean distance, Pearson’s linear correlation coefficient and coefficient of determination (R^2^) were calculated for the neighboring electrodes. A frequency band specific analysis can be found in Supplementary Tables 6, 7 in the Supplementary material.

## 3 Results

**FIGURE 4:**
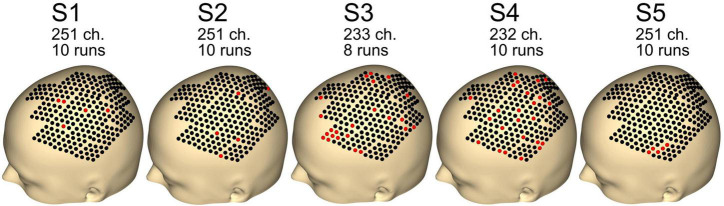
Number of channels and runs used for each subject. Other channels and runs were excluded because of artifacts in the data. Black dots indicate good channels. Red dots indicate channels that were rejected.

Figure 4 shows the remaining channels and runs after the preprocessing step. Data quality was good, with just five channels removed for subjects S1, S2, and S5. For subjects S3 and S4, 23 and 24 channels were removed. For subject S3, two runs were rejected, whereas none were rejected for the other subjects (S1, S2, S4, and S5).

### 3.1 Topography plots

**FIGURE 5:**
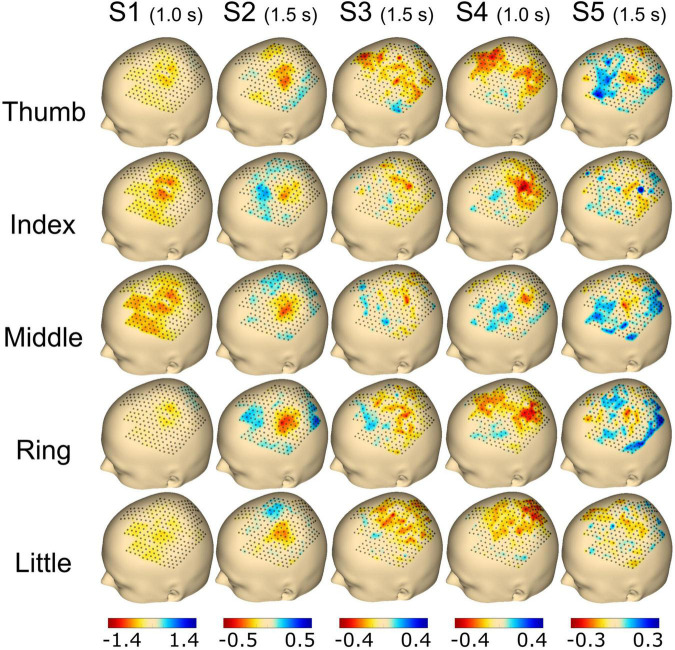
Topography event-related desynchronization/synchronization (ERD/S) plots calculated using the beta frequency band (13–25 Hz) for individual finger movements (rows) from the respective subjects (columns). The selected time point for the calculation is noted in brackets adjoining the subject name. The range of ERD/S was individually set per subject, which is indicated by the range of the color bar on the bottom of each subject’s column and reflects dB.

**FIGURE 6:**
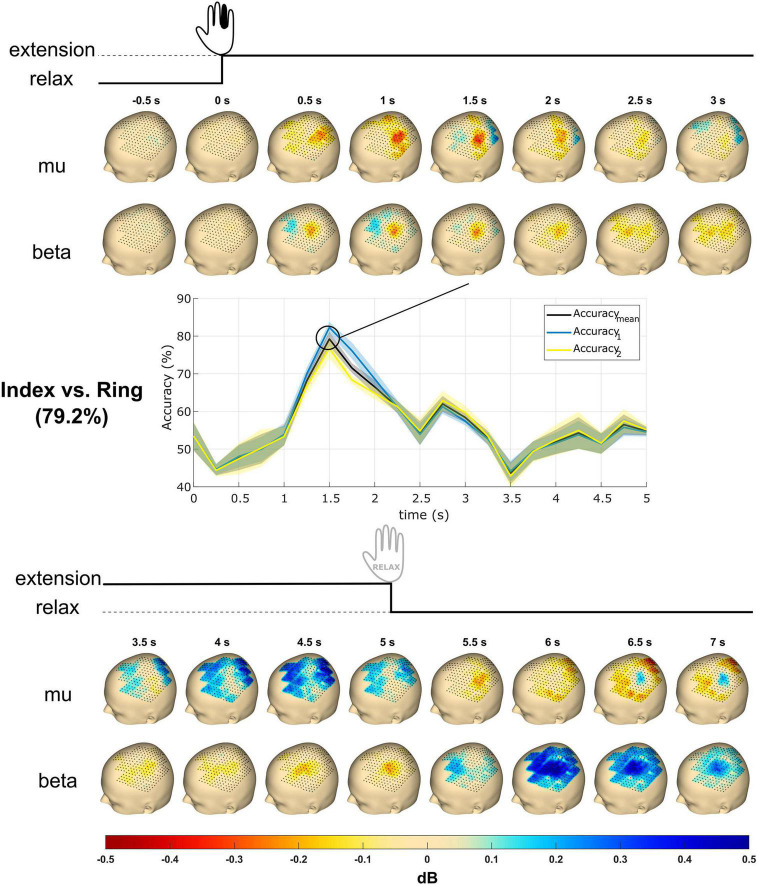
Topography event-related desynchronization/synchronization (ERD/S) plots and time courses of accuracy from subject S2. The subject received the instruction to extend a single finger at cue (0 s), performed the extension, and maintained it until 5 s post-cue. The topography plots were generated using the mu (8–12 Hz) and beta (13–25 Hz) frequency bands with all finger trials averaged. Values reflect dB. The accuracy time courses were obtained using linear support vector machine (SVM) for mu and beta band power features in mean and SD. The classification accuracy reaches a maximum of about 80% at 1.5 s. Accuracy_mean_ is the classification accuracy, whereas Accuracy_1_ and Accuracy_2_ are the accuracies of the respective classes. The most significant accuracy graph was shown here and compared with topography plots.

Event-related desynchronization/synchronization (ERD/S) activation is shown in Figures 5, 6. Every single electrode of the total 256 electrode locations was used to plot the corresponding activation value and interpolated on the scalp of the 3D head model to generate the heatmaps. Bad channels (see Figure 4) were set to 0 for heatmap interpolation.

For Figure 5, the time point selected to plot the ERD/S activation was chosen individually for each subject with either 1.0 s (S1 and S4) or 1.5 s (S2, S3, and S5) post-cue. Finally, the average activity from all trials of the selected time point was plotted for each finger and subject, respectively. For subject S1, we found a clear spot around C3 for the thumb, index, and middle finger extension covering ∼30 electrodes. The activation was smaller for the ring and little finger movements. Subject S2 showed a focal activation spot for all fingers, whereas sparse activation was observed for subjects S3 and S4. Subject S5 had a clear spot for the thumb, index, middle, and ring finger, whereas the little finger showed less activation (see Figure 5).

Figure 6 shows the topography plots obtained from the ERD/S calculation using the mu and beta frequency band features from subject S2 in the time window from 0.5 s pre-cue to 7 s post-cue. A total of 16 subplots are generated with 0.5 s time steps for each frequency band. The mu ERD occurs from 0.5 to 2.5 s and is followed by an ERS from 3.5 to 5 s. The beta ERD occurs from 0.5 to 5.0 s and terminates with an ERS from 6 to 7 s.

**FIGURE 7:**
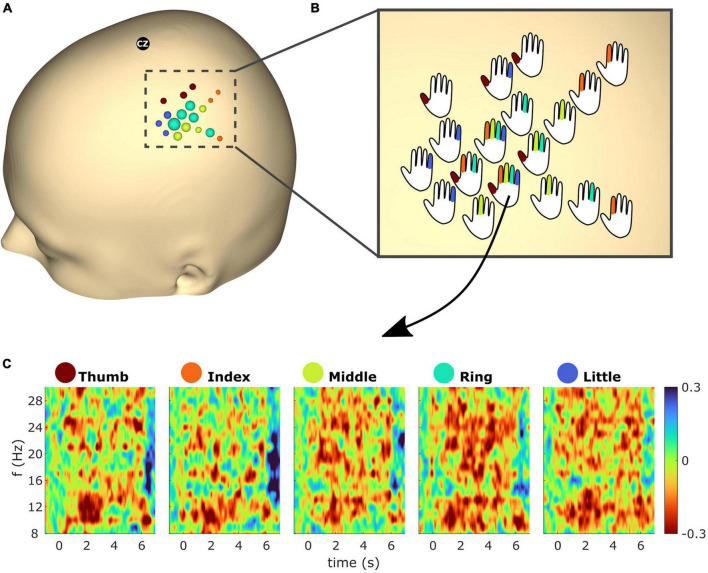
**(A)** Event-related desynchronization/synchronization (ERD/S) bubble plot for all finger movements of subject S2 based on beta (13–25 Hz) frequency band features. Each finger represents a color: thumb (red), index (orange), middle (green), ring (turquoise), and little (blue). The bubbles are scaled according to the mean ERD/S calculated from 1 to 2 s post cue for the average trial in dB. For each finger the ∼6 channels with the greatest ERD were selected (see section “2.4.3” for details). For each electrode position, bubbles were color-coded according to the finger with the greatest ERD. The radius of the bubbles reflects the ERD magnitudes. **(B)** Bubbles from panel **(A)** were replaced with hand schematics. Fingers of each schematic were colored if the electrode was part of the subset which showed the greatest ERD of the respective finger. For electrode No. 125 (see arrow) this was the case for all fingers. **(C)** Time-frequency maps showing the average trial for electrode No. 125 and all finger extensions from subject S2.

Figure 7 shows spatiotemporal ERD/S dynamics of subject S2. The bubble plot shows the ERD/S for all finger extensions (see Figure 7A). As the ∼30 selected electrodes showed overlap across fingers a one vs. all approach was employed, resulting in 18 electrodes being color-coded according to the finger with the greatest ERD. The radius of the bubbles reflects the ERD magnitude. In order to superimpose the overlapping fingers, the bubbles were replaced by hand schematics representing whether the electrode exceeds the threshold (see Figure 7B). Figure 7C shows the temporal ERD/S dynamics for all finger extensions. The time-frequency maps reflect the ERD/S for the electrode which showed the greatest ERD (i.e., within the top 2.5% greatest ERD across all electrodes) from all five finger extensions.

### 3.2 Classification

Table 1 shows the maximum classification accuracies obtained using the linear SVM model and mu and beta band power features. All classification pairs for subject S1 performed above chance (*P* < 0.05). For subjects S2, S3, and S4, 80% of the classification pairs performed above chance level. Finally, for subject S5, 70% of the classification pairs performed above chance level. The highest classification accuracy for subject S1 was 85.4% for middle vs. ring finger. Subject S2 reached 79.2% for index vs. ring finger. Subject S3 achieved 65.0% for thumb vs. middle. Subject S4 reached 71.5% for middle vs. ring, and subject S5 had 66.2% for thumb vs. ring. Finger pairs which include the ring finger generally showed greater classification accuracies than the others. Tasks classifying middle vs. ring finger performed the best overall at 70.6 (9.4)% in mean (SD).

**TABLE 1:** Model performance (classification accuracy) obtained using a linear support vector machine (SVM) in a 10-times of 10-fold CV framework for mu (8–12 Hz) and beta (13–25 Hz) band power features.

Classification	S1		S2		S3		S4		S5		mean (SD)
	mean (SD)	*P*	mean (SD)	*P*	mean (SD)	*P*	mean (SD)	*P*	mean (SD)	*P*		
Thumb vs. Index	62.5 (3.3)	**0.016**	65.4 (2.6)	**0.003**	67.4 (2.3)	**0.016**	58.0 (3.4)	0.081	63.0 (3.4)	**0.048**	63.3 (3.5)
Thumb vs. Middle	65.4 (2.2)	**0.008**	58.4 (3.5)	0.062	**69.4 (2.2)**	**0.016**	65.1 (1.4)	**0.013**	61.8 (2.3)	**0.048**	64.0 (4.1)
Thumb vs. Ring	82.1 (1.3)	**<0.001**	68.1 (1.8)	**0.003**	67.9 (3.6)	**0.016**	64.5 (2.0)	**0.013**	**66.2 (2.3)**	**0.030**	69.8 (7.1)
Thumb vs. Little	66.9 (1.1)	**0.002**	62.0 (1.7)	**0.039**	62.2 (3.3)	**0.046**	60.2 (2.6)	**0.048**	57.6 (2.3)	0.126	61.8 (3.4)
Index vs. Middle	63.2 (4.4)	**0.013**	56.5 (4.0)	0.170	60.6 (2.0)	**0.046**	65.6 (1.5)	**0.013**	55.1 (2.2)	0.213	60.2 (4.4)
Index vs. Ring	78.8 (1.9)	**<0.001**	**79.2 (1.9)**	**<0.001**	59.6 (2.9)	0.084	63.9 (2.1)	**0.013**	61.3 (1.9)	**0.048**	68.6 (9.7)
Index vs. Little	67.1 (2.9)	**0.004**	62.5 (2.5)	**0.010**	56.5 (3.1)	0.217	66.0 (3.2)	**0.009**	56.8 (2.3)	0.126	61.8 (5.0)
Middle vs. Ring	**85.4 (1.2)**	**<0.001**	71.4 (2.9)	**<0.001**	62.0 (2.8)	**0.049**	**71.5 (1.6)**	**0.002**	62.8 (2.3)	**0.048**	**70.6 (9.4)**
Middle vs. Little	66.6 (2.9)	**0.003**	61.8 (2.4)	**0.038**	63.9 (2.6)	**0.036**	60.2 (2.3)	0.060	60.7 (2.3)	**0.048**	62.6 (2.6)
Ring vs. Little	68.8 (1.8)	**0.001**	68.1 (1.9)	**0.003**	67.0 (2.9)	**0.016**	61.6 (3.4)	**0.048**	60.5 (2.4)	**0.048**	65.2 (3.9)
**mean (SD)**	70.7 (8.2)		65.3 (6.7)		63.7 (4.2)		63.7 (3.9)		60.6 (3.3)		**64.8 (6.3)**

Accuracies are reported in percent and as mean (SD). Each subject’s *P*-values are corrected for multiplicity using the Benjamini-Hochberg procedure. The highest accuracies for each subject are bold. Significant *P*-values (*P* <0.05) are bold.

The classification accuracy decreased significantly when using the 10–10 and extended 10–10 system electrode subsets compared to the uHD EEG system (*P* < 0.0001, respectively). Detailed classification results are provided in the Supplementary material, where Supplementary Table 4 shows the results using the 10–10 system subset, and Supplementary Table 5 shows the model performance using the extended 10–10 system. Specifically, using the 10–10 and extended 10–10 system electrode subsets resulted in a classification accuracy decrease of 6.9 [4.6; 10.0]% and 5.9 [0.6; 9.7]% in median [IQR], respectively over all subjects and pairwise classifications.

### 3.3 Correlation between neighboring electrodes

Table 2 shows the Euclidean distance, Pearson’s linear correlation coefficient and *R*^2^ value for neighboring electrodes in median [IQR] for the 10–10, extended 10–10 and uHD EEG system, respectively. The median correlation between neighboring electrodes increases with their median Euclidean distance decreasing.

**TABLE 2:** Euclidean distance, Pearson’s linear correlation coefficient (*r*) and coefficient of determination (*R*^2^) for neighboring electrodes for the 10–10, extended 10–10 and ultra-high-density electroencephalography (uHD EEG) system.

EEG system	Euclidean distance (mm)	*r*	*R* ^2^
	Median [IQR]	Median [IQR]	Median [IQR]
10–10	35.4 [30.9; 38.0]	0.43 [0.14; 0.61]	0.18 [0.04; 0.37]
Ext. 10–10	23.6 [17.6; 25.2]	0.57 [0.37; 0.72]	0.32 [0.14; 0.51]
uHD	8.6 [8.5; 8.7]	0.66 [0.52; 0.79]	0.44 [0.27; 0.62]

Metrices are reported in median [IQR].

## 4 Discussion

### 4.1 Topography plots

We could clearly show that the better spatial resolution of the uHD EEG system compared to standard EEG systems allows a more detailed understanding of temporal and spatial dynamics. The superimposed plot for all five fingers allows a detailed investigation of the 18 sensor locations with highest activation for all finger movements in subject S2 (see Figure 7). This activation area was approx. five times six columns wide, which resulted in 45 mm times 54 mm coverage surface. In standard EEG recordings, only two to four electrodes would overlay this area.

Electrocorticography (ECoG) electrode grids typically have a center-to-center distance of 10 mm, which is even larger than the uHD EEG system with 8.6 mm. However, the big advantage of ECoG remains–the possibility to capture high gamma or even ultra-high gamma frequencies, which is difficult with uHD EEG due to skin, bones, and fluids attenuating the signal captured on the scalp.

The electrodes seen in Figure 7A represent a focal spot overlaying the sensorimotor cortex (Ejaz et al., 2015). Across the five fingers, overlap in electrodes which showed the greatest beta ERD can be observed. This is in line with fMRI studies which reported overlapping areas throughout the primary motor cortex (M1) hand region (Sanes et al., 1995; Schieber, 1999; Ejaz et al., 2015) which are likely due to co-existence of somatotopic- and action maps (Graziano and Aflalo, 2007; Huber et al., 2020). Note that this is not the case for primary somatosensory cortex (S1) which has more distinct somatotopic organization (Schweisfurth et al., 2014; Huber et al., 2020; O’Neill et al., 2020). However, as the subjects’ exact central sulcus location is unknown in this work we could not determine which electrodes are located over M1 or S1. Furthermore the current work focused on the low-frequency bands (mu and beta), whereas for cortical mapping high frequency components are used [e.g., Kapeller et al. (2018)].

For both the time-frequency maps and the heatmap topographies (see Figures 6, 7) one can observe desynchronization with movement on- and offset. In fact, this phenomenon was discovered for all subjects and is consistent with findings in literature (Cassim et al., 2000; Sun et al., 2016). The observed ERD increases at the end of sustained movements, which may be associated with the cortical preparation before movement termination. ECoG studies also report similar increases in high-gamma activity for movement onset and termination (Flint et al., 2016). The decrease in ERD around 3–4 s post-cue matches the findings of Cassim et al. (2000) that mu and beta power return to baseline values in sustained movements, which may be associated with requiring less muscular activity and concentration (Bernard et al., 2002; Cramer et al., 2002). Indeed, this could explain why ERD is much more sustained (around 3–4 s post-cue) for the ring finger, as a controlled ringer finger extension requires more effort and concentration. Finally, post-movement beta rebounds (PMBR) can be observed for both heatmap topographies and time-frequency maps after movement termination at roughly 6.5 s. While PMBR is a well-known phenomenon, its function(s) is still not fully understood [see Hervault et al. (2021) for a recent investigation].

This first study was done with real finger movements without real-time feedback. Such non-feedback experiments prove the system’s feasibility to be used for the intended use, finger movement detection in this case. Studies from Guger, Pfurtscheller, and many others have shown that feedback change brain patterns and make them much more stable (Guger et al., 2000; Pfurtscheller, 2001; Miller et al., 2010). Therefore, our next studies will include feedback. The disadvantage of real movement studies is that brain activation terminates quickly as soon as no movement is performed. On the other hand, if motor imagery is used, subjects are able to activate the brain much longer (Gruenwald et al., 2017).

In some subjects, more bad channels had to be excluded from further processing. This happened because the adhesive did not fixate the grids strongly enough. In future experiments, a stronger adhesive will be used to ensure that more channels show good signals. This will also ensure that fewer trials will show artifacts.

### 4.2 Classification

We could show that single fingers can be discriminated with the uHD EEG system. Peak accuracy ranged from 85.4(S1), 79.2(S2), 69.4(S3), 71.5(S4) to 66.2(S5). Mean accuracies of 70.7(S1), 65.3(S2), 63.7(S3), 63.7(S4), and 60.6(S5) are in the range of other motor movement studies without feedback (Quandt et al., 2012; Bera et al., 2019). The difference is that now we are classifying single fingers, not left/right motor imagery. As stated in section “1,” the possibility of distinguishing between finger movements was sufficiently shown by ECoG studies (Kubánek et al., 2009; Hotson et al., 2016; Gruenwald et al., 2019) but few for EEG with limited performance (Liao et al., 2014; Stankevich et al., 2016; Bera et al., 2019). Essential to push the classification accuracy much further is to include feedback which will be done in the subsequent study.

The observation that finger pairs that include the ring finger have greater classification accuracy than others could be due to the ring finger extension requiring more effort than the other finger extensions. Reasonably, one may expect thumb vs. little finger classification accuracy to be the best as the somatotopic distance between the two fingers is large according to fMRI (e.g., Berlot et al., 2019) and ECoG studies (e.g., Miller et al., 2012). In contrast, we observed lower performance for this finger pair with a classification accuracy of 61.8 (3.4)% across subjects. However, this phenomenon may be explained by the fact that blood oxygenation level-dependent (BOLD) imaging or high-gamma activity is typically used for somatotopic investigations. Importantly, BOLD and high-gamma activity are related (Logothetis et al., 2001; Mukamel et al., 2005; Niessing et al., 2005; Engell et al., 2012) and have better somatotopic characteristics than low-frequency (i.e., mu and beta) band power (Miller et al., 2007, 2012). This may explain that somatotopic distance between fingers did not positively impact the observed classification accuracies.

The obtained classification accuracies [64.8 (6.3)% on average] can only be indirectly compared to Bera et al. (2019) and Liao et al. (2014). While as they also performed pairwise classification of finger movements, they used different features and classification models. Specifically, Bera et al. (2019) conducted finger extension tasks including thumb, index, and middle fingers but used CSPs, LDA and Extra Trees Classifiers for classification, achieving an overall average accuracy of 60.3% with two preceding training days. Liao et al. (2014) conducted two repetitive finger flexions and extensions tasks during 2 s. They used a SVM with a radial basis kernel function and achieved an average classification accuracy of 58.6 and 57.7% using mu and beta band power features, respectively. Note that since the exact experimental protocol (e.g., finger movements, number of trials) and analysis procedures are different, the results should not be directly compared.

Classification models based on the uHD EEG system outperformed models based on electrode subsets reflecting the 10–10 and extended 10–10 system by roughly 6 and 7% classification accuracy on median over all subjects and pairwise classifications.

### 4.3 Correlation between neighboring electrodes

Unsurprisingly, the correlation between neighboring electrodes increases with decreasing Euclidean distance (see Table 2). However, the observed correlations for the uHD EEG system are still far from perfect correlations. Furthermore, the relationship between Euclidean distance and correlation is not linear, which can be shown using the *R*^2^ value. In the current context, *R*^2^ quantifies the percentage of variance explained for one electrode given its neighboring electrode and is thus a measure of similarity. Specifically, when comparing the extended 10–10 and uHD EEG system the Euclidean distance roughly decreases by a factor of 2.7, while the amount of similarity between neighboring electrodes increases by 12% on median. Importantly, this increase in similarity is less than going from 10–10 to extended 10–10 system for which a median increase of 14% can be observed and in this case Euclidean distance only decreases by a factor of 1.5. Notably, the amount of unexplained variance (1-*R*^2^) is still 56% on median for neighboring electrodes for the uHD EEG system.

### 4.4 Limitations and outlook

In this study pairwise finger classification (i.e., binary classification) was performed instead of multiclass classification, where all five fingers are classified at once. The latter approach would allow for online (i.e., real-time) feedback provided by the hand avatar, which likely increases subjects’ engagement and classification accuracy. However, for a multiclass classifier to work reasonably well, pairwise classifications would need to reach high classification accuracies (e.g., > 85%). Achieving higher classification accuracies is likely possible as different features, feature/electrode selection methods and machine learning models can be employed. Indeed, methods such as Riemannian spatial patterns (Larzabal et al., 2021) may also be applied to gain insights in which electrodes are most important for decoding individual finger movements.

Topography ERD/S plots benefit from the uHD EEG technology, as seen in this study. Michel and Brunet (2019) stated that source reconstruction profits from high-density EEG technologies. The larger number of electrode points and the higher spatial density provide the necessary information. Therefore, the domain of source localization with procedures such as Low-Resolution Brain Electromagnetic Tomography Analysis (LORETA) could take advantage of the higher spatial resolution and electrode numbers. However, source reconstruction methods were not explored in the current work but should be explored in future work.

Adopting spatial mapping of the central sulcus using fMRI scans could provide important neuroanatomical information for the exact allocation of the electrode positions. Thus, more detailed assignment of acquired signals to the real spatial positions of the electrodes themselves can be done.

## Data availability statement

The datasets presented in this study can be found in online repositories. The names of the repository/repositories and accession number(s) can be found below: https://osf.io/4dwjt/?view_only=d23acfd50655427fbaae381a17cbfbcc.

## Ethics statement

The studies involving human participants were reviewed and approved by Institutional Review Board of Korea Advanced Institute of Science and Technology (KH2018-127). The patients/participants provided their written informed consent to participate in this study.

## Author contributions

HL, S-HJ, CG, and H-SP conceived the study. HP provided scientific input. HL and S-HJ designed and conducted experiments. MJ and LS created topography plots. SS performed data analyses. LS, S-HJ, and SS interpreted the result. HL, LS, S-HJ, and SS wrote and edited the manuscript. All authors contributed to the article and approved the submitted version.
