# Supplementary material for: Individual finger movement decoding using a novel ultra-high-density electroencephalography-based brain-computer interface system
Source: Front Neurosci. 2022 Oct 19;16:1009878. doi: 10.3389/fnins.2022.1009878 (PMC9627315; doi:10.3389/fnins.2022.1009878)
Supplement: Supplementary file 1 [file Data_Sheet_1.pdf]

frontiers

Supplementary Material

1 Supplementary Data

The raw EEG data and cue information are publicly available and described in the manuscript.

2 Supplementary Figures and Tables

2.1 High-gamma Activity Features

Next to mu and beta band features, high-gamma band power features were also investigated. **Figure S1** shows the processing pipeline for these features. Note that raw EEG recordings were not downsampled, and the window lengths of the band power estimation were reduced as the high-gamma features are assumed to have faster characteristics than the mu and beta features. Furthermore, an optional whitening filter was introduced and applied to each channel (Oppenheim and Schafer, 2010). This was done to equalize the 1/f-shape of the spectrum (Gruenwald et al., 2019). Specifically, the coefficients of a 10th-order auto-regressive model were estimated using the Yule-Walker method (*aryule*), which were then used as the filter coefficients of a finite-impulse response filter. For more information see Gruenwald et al. (2019).

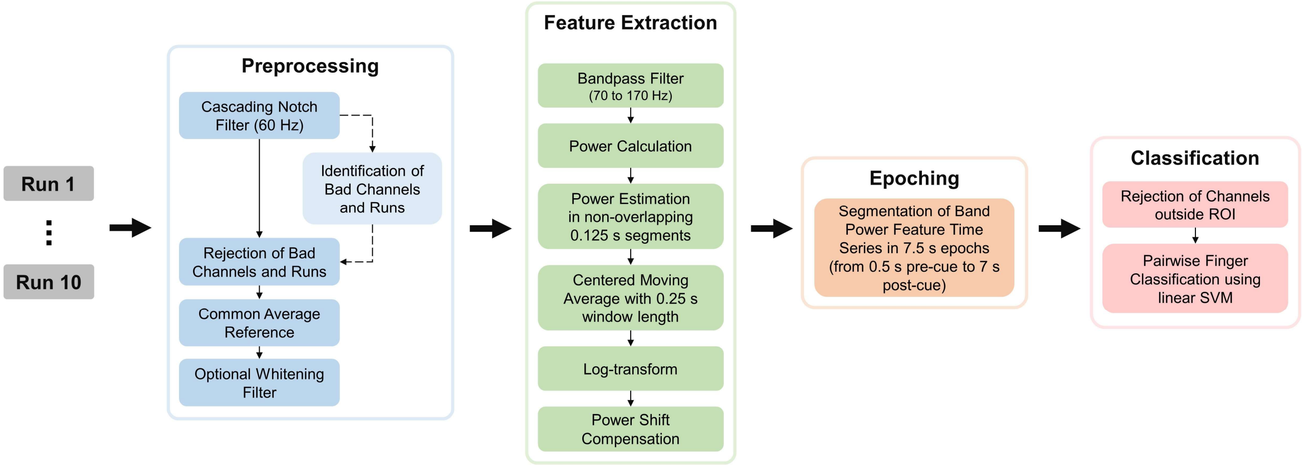

Figure S1 | EEG processing pipeline for high-gamma features consisting of Preprocessing, Feature Extraction, Epoching, and Classification. “Identify Bad Channels and Runs” returns only the information which channel(s) and run(s) are bad (dashed arrow) while leaving the EEG data (solid arrows) unchanged. Form of data after each process is visualized in dashed box.

High-gamma feature topography plots are not shown as no topographical information could be observed independent of the optional whitening filter. **Tables S1** and **S2** show the pairwise classification results for the 5 subjects. Note that no practical differences between the usage of the optional whitening filter could be observed. Model performance was generally subpar when using high-gamma features.

Table S1 | Model performance (classification accuracy) obtained using a linear SVM in a 10-times of 10-fold CV framework for high-gamma band power features without optional whitening. Accuracies are reported in percent and as mean(SD). Each subject's P-values are corrected for multiplicity using the Benjamini-Hochberg procedure. The highest accuracies for each subject are bold. Significant P-values (P<0.05) are marked in bold.

Classification	S1		S2		S3		S4		S5		mean(SD)
	mean(SD)	P	mean(SD)	P	mean(SD)	P	mean(SD)	P	mean(SD)	P	
Thumb vs. Index	65.7(1.2)	**0.019**	61.4(2.8)	**0.012**	68.3(2.0)	**0.007**	60.3(1.9)	**0.047**	56.8(2.0)	0.205	62.5(4.5)
Thumb vs. Middle	64.3(2.2)	**0.019**	64.8(2.0)	**0.005**	**69.4(1.5)**	**0.005**	64.5(3.6)	**0.024**	**65.1(2.8)**	**0.028**	65.6(2.1)
Thumb vs. Ring	63.1(3.1)	**0.019**	**70.2(1.6)**	**0.002**	69.1(1.9)	**0.007**	63.1(1.3)	**0.025**	59.3(2.4)	0.061	65.0(4.6)
Thumb vs. Little	58.5(4.1)	0.060	67.4(1.7)	**0.002**	64.2(2.6)	**0.018**	58.5(2.5)	0.078	60.2(2.7)	0.060	61.8(3.9)
Index vs. Middle	60.9(2.1)	**0.048**	66.7(1.9)	**0.002**	63.4(3.6)	**0.027**	60.1(2.6)	**0.044**	64.1(3.4)	**0.028**	63.0(2.6)
Index vs. Ring	60.6(1.3)	**0.038**	66.4(2.1)	**0.005**	62.4(2.3)	**0.031**	**71.8(3.2)**	**0.006**	59.4(2.5)	0.129	64.1(5.0)
Index vs. Little	62.2(3.2)	**0.038**	68.6(2.7)	**0.002**	63.3(2.1)	**0.021**	62.7(3.0)	**0.025**	62.5(2.2)	**0.043**	63.9(2.7)
Middle vs. Ring	61.5(4.0)	**0.027**	62.9(3.0)	**0.012**	62.9(2.8)	**0.021**	62.3(1.8)	**0.025**	63.2(2.1)	**0.043**	62.6(0.7)
Middle vs. Little	**70.4(2.3)**	**<0.001**	67.8(2.4)	**0.002**	66.1(1.9)	**0.007**	63.7(2.3)	**0.024**	61.2(1.5)	0.060	**65.8(3.6)**
Ring vs. Little	63.5(2.5)	**0.019**	67.9(2.1)	**0.002**	61.9(2.1)	**0.035**	61.8(3.0)	**0.025**	59.6(3.3)	0.060	62.9(3.1)
**mean(SD)**	63.1(3.3)		66.4(2.7)		65.1(2.9)		62.9(3.6)		61.1(2.6)		**63.7(3.4)**

Table S2 | Model performance (classification accuracy) obtained using a linear SVM in a 10-times of 10-fold CV framework for high-gamma band power features with optional whitening. Accuracies are reported in percent and as mean(SD). Each subject's P-values are corrected for multiplicity using the Benjamini-Hochberg procedure. The highest accuracies for each subject are bold. Significant P-values (P<0.05) are marked in bold.

Classification	S1		S2		S3		S4		S5		mean(SD)
	mean(SD)	P	mean(SD)	P	mean(SD)	P	mean(SD)	P	mean(SD)	P	
Thumb vs. Index	63.7(3.0)	**0.017**	63.9(2.6)	**0.009**	66.3(2.1)	**0.022**	62.2(2.1)	**0.042**	59.9(2.2)	0.097	63.2(2.4)
Thumb vs. Middle	60.7(2.9)	0.055	65.2(2.1)	**0.005**	66.8(1.5)	**0.012**	62.8(2.4)	**0.034**	58.9(2.4)	0.097	62.9(3.2)
Thumb vs. Ring	61.8(2.0)	**0.032**	**70.8(2.3)**	**0.002**	**68.4(2.3)**	**0.012**	64.3(2.4)	**0.034**	60.3(1.9)	0.055	**65.1(4.4)**
Thumb vs. Little	57.9(3.3)	0.114	68.8(2.0)	**0.002**	60.1(3.0)	0.054	57.1(4.4)	0.106	61.9(0.9)	0.055	61.2(4.7)
Index vs. Middle	65.4(3.0)	**0.016**	64.8(2.8)	**0.009**	61.2(1.2)	**0.037**	64.0(1.7)	**0.034**	60.1(1.9)	0.055	63.1(2.3)
Index vs. Ring	58.9(3.5)	**0.048**	66.9(2.0)	**0.005**	62.2(3.8)	**0.025**	**67.9(2.4)**	**0.020**	58.3(1.5)	0.097	62.8(4.4)
Index vs. Little	**66.0(2.2)**	**0.016**	67.9(2.3)	**0.002**	60.3(2.7)	0.060	59.9(2.5)	0.056	63.0(1.8)	**0.026**	63.4(3.5)
Middle vs. Ring	64.7(3.8)	**0.016**	61.0(3.8)	**0.034**	61.8(2.3)	**0.037**	62.2(3.4)	**0.034**	**64.4(1.8)**	**0.026**	62.8(1.6)
Middle vs. Little	64.9(2.0)	**0.016**	64.6(4.1)	**0.008**	62.0(2.6)	**0.037**	62.5(1.7)	**0.034**	59.3(2.3)	0.097	62.7(2.3)
Ring vs. Little	60.2(2.7)	0.052	69.8(1.5)	**0.005**	62.0(2.8)	**0.037**	63.5(2.5)	**0.037**	60.8(2.1)	0.061	63.3(3.9)
**mean(SD)**	62.4(2.9)		66.4(3.0)		63.1(2.9)		62.6(2.8)		60.7(1.9)		**63.0(3.2)**

2.2 Power Shift Compensation

As described in section 2.4.2 in the manuscript, a power shift compensation (PSC) was used to eliminate slow drifts in band power features over time. **Figure S2** shows an example of the resulting PSC signal if one applies a causal moving average filter with a window length of 25 s to the beta band power features of subject S2 using two channels and two different runs. Note how the PSC signal for channel 168 shows great variation over the relatively short period of 3 minutes. On the other hand, this phenomenon is less pronounced for channel 173. Importantly, this phenomenon is not exclusive to the ultra-high-density EEG system used but can also be observed in standard EEG recordings.

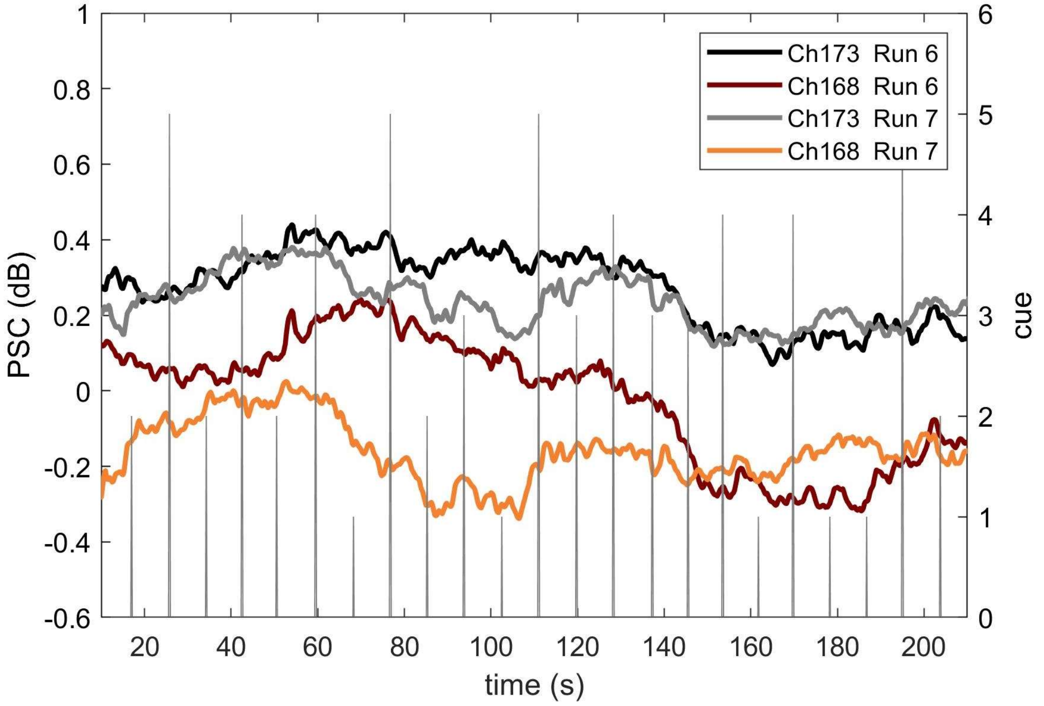

Figure S2 | Power Shift Compensation (PSC) signals for subject S2 for two channels and two runs, which were obtained by applying a causal moving average filter to the beta band power features. The dirac-like impulses are the cues for the 7 runs (1 to 5 reflecting thumb to little finger).

2.3 Leave-one-run-out CV

As described in section 2.4.4 in the manuscript, one may also perform a leave-one-run-out CV in which one trains the model on all but one run (i.e., recording) and then uses this remaining run as test data to obtain the model performance. The performances (i.e., mean classification accuracy over all folds) obtained using this framework are shown in **Table S3** and are similar to the ones obtained using 10-times of 10-fold CV. Note that subject S3's results are only based on 8 runs as 2 runs were rejected.

Table S3 | Model performance (classification accuracy) obtained using a linear SVM in a leave-one-run-out CV framework for mu (8 – 12 Hz) and beta (13 – 25 Hz) band power features. Accuracies are reported in percent and mean or mean(SD) as applicable. The highest accuracies for each subject are bold.

**Classification**	**S1**	**S2**	**S3**	**S4**	**S5**	**mean(SD)**
Thumb vs. Index	62.0	63.0	67.5	61.0	**66.0**	63.9(2.7)
Thumb vs. Middle	63.0	60.0	68.7	68.0	65.0	64.9(3.6)
Thumb vs. Ring	83.0	70.0	67.5	67.0	64.0	70.3(7.4)
Thumb vs. Little	69.0	63.0	63.7	63.0	63.0	64.3(2.6)
Index vs. Middle	61.0	59.0	62.5	69.0	60.0	62.3(4.0)
Index vs. Ring	80.0	**78.0**	61.3	67.0	64.0	70.1(8.4)
Index vs. Little	68.0	63.0	55.0	67.0	58.0	62.2(5.6)
Middle vs. Ring	**85.0**	72.0	61.3	**71.0**	64.0	**70.7(9.2)**
Middle vs. Little	64.0	61.0	**68.8**	62.0	65.0	64.2(3.0)
Ring vs. Little	68.0	70.0	66.3	64.0	62.0	66.1(3.2)
**mean(SD)**	70.3(9.0)	65.9(6.2)	64.3(4.4)	65.9(3.2)	63.1(2.5)	**65.9(5.9)**

2.4 Electrode Subsets

Electrode subsets reflecting the 10-10 and extended 10-10 system were selected in order to investigate how the additional electrodes introduced by the uHD EEG system change classification performance and correlation between neighboring electrodes. The electrode subsets were generated by selecting single uHD electrodes which best fit the respective 10-10 and extended 10-10 system positions (see **Figure S3**).

Classification results using these electrode subsets are shown in **Tables S4** and **S5**.

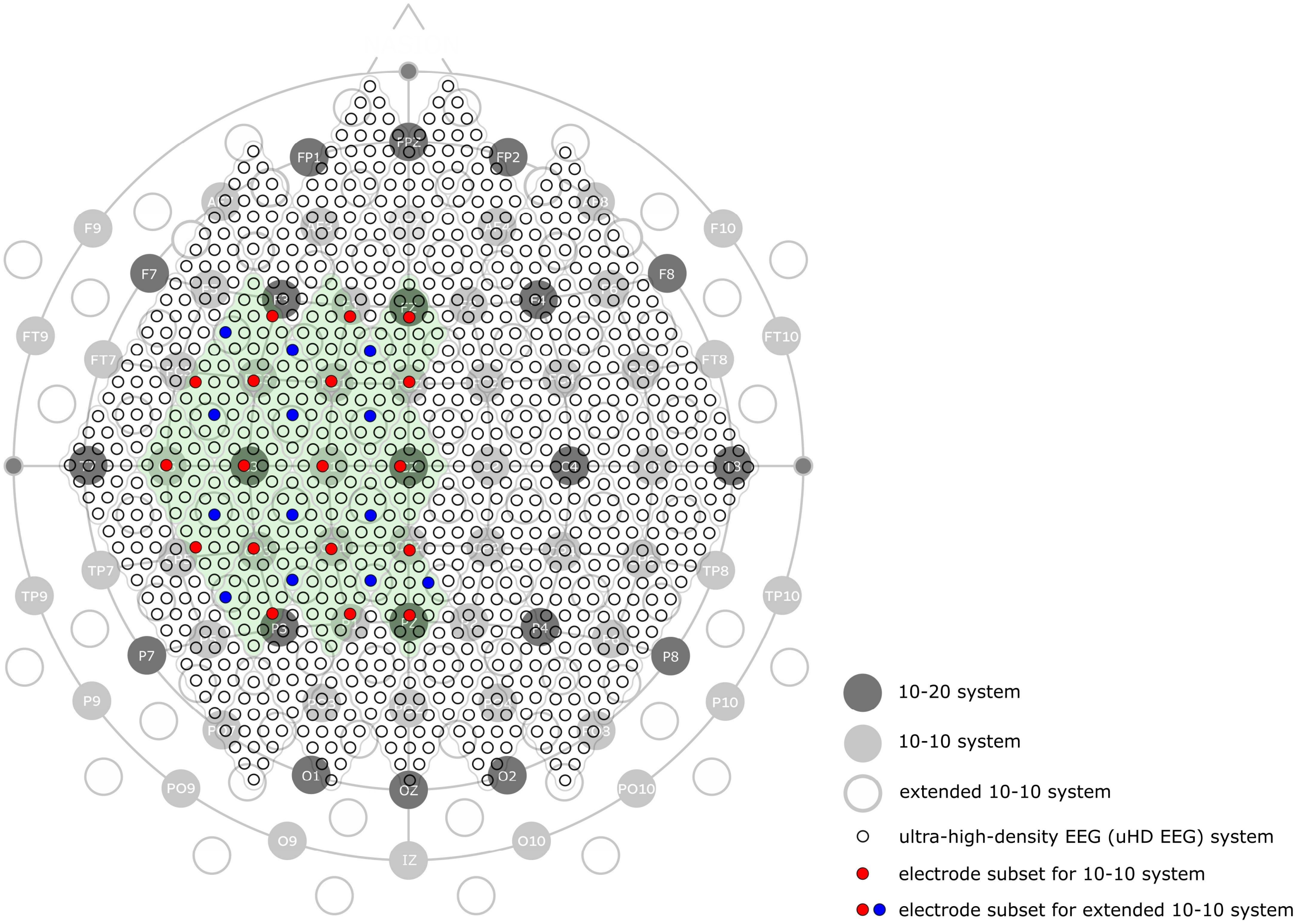

Figure S3 | Electrode subsets for the 10-10 and extended 10-10 system

Table S4 | Model performance (classification accuracy) obtained using a linear SVM in a 10-times of 10-fold CV framework for mu (8 – 12 Hz) and beta (13 – 25 Hz) band power features based on the electrode subset for the 10-10 system. Accuracies are reported in percent and mean(SD). The highest accuracies for each subject are bold.

Classification	S1	S2	S3	S4	S5	mean(SD)
Thumb vs. Index	53.4(2.1)	54.5(3.2)	51.5(2.4)	61.4(2.1)	56.5(2.3)	55.4(3.8)
Thumb vs. Middle	52.7(4.4)	51.9(3.2)	53.5(2.8)	60.4(1.9)	52.8(3.9)	54.3(3.5)
Thumb vs. Ring	72.7(2.2)	66.1(2.7)	61.4(2.3)	56.2(2.5)	61.6(1.4)	**63.6(6.2)**
Thumb vs. Little	49.4(2.1)	53.2(2.4)	47.4(4.0)	49.1(3.0)	56.3(2.9)	51.1(3.6)
Index vs. Middle	56.6(2.4)	49.3(2.7)	**63.5(2.4)**	64.2(2.5)	50.5(2.9)	56.8(7.0)
Index vs. Ring	74.2(1.5)	**72.6(1.4)**	56.0(2.8)	55.9(1.8)	53.2(1.9)	62.4(10.1)
Index vs. Little	67.0(1.8)	55.2(2.1)	52.1(2.3)	53.0(3.1)	46.9(2.9)	54.8(7.4)
Middle vs. Ring	**74.8(1.8)**	65.0(2.5)	59.5(2.0)	58.8(3.9)	58.1(2.2)	63.3(7.0)
Middle vs. Little	58.6(3.7)	47.3(2.1)	56.1(2.7)	**65.5(2.7)**	50.7(3.5)	55.6(7.1)
Ring vs. Little	55.0(3.0)	66.2(2.4)	61.8(3.9)	56.1(3.9)	**62.5(2.1)**	60.3(4.6)
**mean(SD)**	61.4(9.8)	58.1(8.6)	56.3(5.2)	58.1(5.0)	54.9(5.0)	**57.8(7.1)**

Table S5 | Model performance (classification accuracy) obtained using a linear SVM in a 10-times of 10-fold CV framework for mu (8 – 12 Hz) and beta (13 – 25 Hz) band power features based on the electrode subset for the extended 10-10 system. Accuracies are reported in percent and mean(SD). The highest accuracies for each subject are bold.

Classification	S1	S2	S3	S4	S5	mean(SD)
Thumb vs. Index	55.9(2.8)	61.3(3.4)	58.4(2.8)	62.1(2.0)	48.3(2.5)	57.2(5.6)
Thumb vs. Middle	51.3(2.1)	57.3(3.0)	**68.8(3.1)**	58.4(2.5)	**61.6(1.7)**	59.5(6.4)
Thumb vs. Ring	73.9(2.2)	64.5(2.4)	49.7(3.3)	62.0(4.2)	58.1(3.0)	61.6(8.9)
Thumb vs. Little	51.2(1.8)	59.4(2.0)	44.4(3.0)	59.6(1.1)	50.0(2.7)	52.9(6.5)
Index vs. Middle	57.2(2.6)	55.1(2.3)	50.8(4.6)	57.4(4.8)	43.3(2.4)	52.7(5.9)
Index vs. Ring	74.6(2.9)	74.3(1.4)	60.5(2.6)	**66.3(3.0)**	55.5(1.9)	**66.2(8.4)**
Index vs. Little	68.2(2.6)	64.1(2.0)	54.7(2.3)	58.8(1.6)	47.1(3.1)	58.6(8.2)
Middle vs. Ring	**75.3(2.0)**	**77.7(2.2)**	56.9(5.2)	59.9(2.3)	56.8(3.0)	65.3(10.3)
Middle vs. Little	53.8(2.0)	62.2(2.1)	51.9(5.0)	52.9(3.8)	60.2(1.8)	56.2(4.7)
Ring vs. Little	62.3(4.1)	70.8(1.5)	57.3(3.7)	56.0(2.0)	60.3(2.7)	61.3(5.8)
**mean(SD)**	62.4(9.8)	64.7(7.4)	55.3(6.7)	59.4(3.7)	54.1(6.4)	**59.2(7.9)**

2.5 Correlation Between Neighboring Electrodes

Additional correlation analyses were performed for specifc frequency bands (delta: 1 – 4 Hz, theta: 1 – 4 Hz, mu/alpha: 8 – 12 Hz, beta: 13 – 25 Hz) as described in section 2.4.5 in the manuscript. This was done, because the spectrum has a 1/f-shape. Therefore, if one filteres between 1 to 30 Hz, lower frequency components (i.e., delta and theta) contribute to the majority of EEG magnitude, compared to "higher" frequency components (i.e., alpha and beta). **Tables S6** and **S7** show the Pearson's linear correlation coefficient and coefficient of determination for the neighboring electrode pairs as median [IQR] for the specific frequency bands.

Table S6 | Pearson's linear correlation coefficient for neighboring electrodes for the respective frequency bands. Values are reported as median [IQR].

EEG System	Pearson's linear correlation coefficient (r)			
	delta	theta	mu	beta
**10-10**	0.40 [0.13; 0.60]	0.45 [0.16; 0.57]	0.49 [0.28; 0.67]	0.34 [0.19; 0.55]
**Ext. 10-10**	0.54 [0.29; 0.72]	0.57 [0.43; 0.68]	0.65 [0.51; 0.78]	0.52 [0.37; 0.67]
**uHD**	0.61 [0.40; 0.78]	0.66 [0.52; 0.77]	0.74 [0.61; 0.85]	0.66 [0.51; 0.78]

Table S7 | Coefficient of determination (R^2) for neighboring electrodes for the respective frequency bands. Values are reported as median [IQR].

EEG System	Coefficient of determination (R^2)			
	delta	theta	mu	beta
**10-10**	0.18 [0.04; 0.36]	0.20 [0.04; 0.33]	0.25 [0.09; 0.45]	0.12 [0.04; 0.30]
**Ext. 10-10**	0.29 [0.08; 0.51]	0.32 [0.19; 0.46]	0.42 [0.26; 0.60]	0.27 [0.14; 0.45]
**uHD**	0.37 [0.17; 0.60]	0.44 [0.27; 0.60]	0.55 [0.37; 0.73]	0.44 [0.26; 0.61]

3 References

Gruenwald, J., Znobishchev, A., Kapeller, C., Kamada, K., Scharinger, J., and Guger, C. (2019). Time-Variant Linear Discriminant Analysis Improves Hand Gesture and Finger Movement Decoding for Invasive Brain-Computer Interfaces. *Front. Neurosci.* 13, 901. doi: 10.3389/fnins.2019.00901.

Oppenheim, A. V., and Schafer, R. W. (2010). *Discrete-Time Signal Processing: International Edition*. 3rd edition. Upper Saddle River, NJ: Pearson.
